# Perspectives on Using Platelet-Rich Plasma and Platelet-Rich Fibrin for Managing Patients with Critical Lower Limb Ischemia After Partial Foot Amputation

**DOI:** 10.25122/jml-2020-0028

**Published:** 2020

**Authors:** Volodymyr Goshchynsky, Bogdan Migenko, Oleg Lugoviy, Ludmila Migenko

**Affiliations:** 1. Department of Surgery, Institute of Postgraduate Education, I. Horbachevsky Ternopil National Medical University, Ternopil, Ukraine.; 2. Second Department of Internal Medicine, I. Horbachevsky Ternopil National Medical University, Ternopil, Ukraine.

**Keywords:** limb revascularization, foot amputation, wound healing, PRGF-platelet-rich growth factor, PRF-platelet-rich fibrin, PRP- platelet-rich plasma

## Abstract

The problem of lower limb preservation with symptoms of critical ischemia, resulting in necrosis of the distal foot portion, remains open. These cases require solving few tactical questions, such as the primary revascularization method, limb-preserving amputation, stimulation of regeneration, and finally, determining the criteria for auto-dermal transplantation.

We analyzed 29 patient cases with critical lower limb ischemia of fourth grade, according to the Fontaine classification (or the sixth category according to Rutherford's classification), who underwent partial foot amputation due to dry gangrene and were threated using PRGF®-ENDORET® platelet-rich plasma and platelet-rich fibrin technology. The control group was comprised of 21 patients who received traditional postoperative wound treatment. All patients went through a combination of transluminal revascularization and platelet-rich plasma to create a “therapeutic” neoangiogenic effect. Indications for these procedures were severe distal arterial occlusion and stenosis.

Using transluminal procedures with platelet-rich plasma therapy improves the blood perfusion to the distal portions of the limb in patients with critical ischemia in a short time, which is an informative diagnostic criterion for wound healing after amputation. Plasmatic membranes create an optimal environment for tissue regeneration, thus reducing the wound closure time using an auto-dermal transplant.

## Introduction

Choosing a proper surgical approach remains a big issue in managing patients with critical lower limb ischemia [1-4]. Direct revascularization is possible only in 30-40% of this patient category [5-6]. Therefore, to treat patients with critical lover limb ischemia, the surgeon chooses one of the two options: limb amputation or attempt to restore limb blood flow [7]. Today, 70 to 80% of amputations are performed due to critical lower limb ischemia. There is a significant decrease in the life level and life expectancy of a post-amputation patient [8-9]. Therefore, limb preservation with blood perfusion renewal using direct or indirect revascularization is a critical task. Therapeutic angiogenesis (bone marrow mononuclear injections, genetic therapy, thrombocyte solutions) are considered to be promising methods of treatment [10-13].

While the tactical and technical aspects of surgical treatment of critical ischemia are well represented, the postamputation wound healing process is not entirely resolved. Naturally, wound healing in this category of patients depends on many factors, such as foot and lower limb blood perfusion quality, which determines the healing process and resistance to local infection. From a practical standpoint, it is critical to reduce the preparation time for auto-dermal transplantation, which will ensure foot support function and patient rehabilitation [4, 15].

The objective of our study was to create clinical recommendations for using platelet-rich plasma (PRP) and platelet-rich fibrin (PRF) for wound healing stimulation after foot amputation patients with critical limb ischemia (CLI).

## Material and Methods

We analyzed 29 cases of patients with fourth grade critical lower limb ischemia, according to R. Fontaine (the sixth category according to the Rutherford classification) who undergone partial foot amputation due to dry gangrene and were threated using PRGF®-ENDORET® PRP and PRF technology. The control group was comprised of 21 patients who underwent traditional postoperative wound treatment. The average age was 56.7 ± 9.3 years, and the patients were all males. For ultrasound diagnostic of arterial vessel disease, a Vivid 3 (General Electric, USA) device with a 5-10 MHz range sensor and standard software were used. A CT scan with 3D reconstruction or a Siemens angiography system was used to locate an atherosclerotic plaque and its length. Using the TASC II classification for sonography and angiography [26], type C was found in 26 patients, and type D in 24 patients. All patients received a combination of transluminal revascularization and PRP with therapeutic neoangiogenesis. Indications for the latter are severe arterial occlusion and stenosis of the distal segments. PRP therapy was performed three weeks prior to the endovascular procedure.

For therapeutic neoangiogenesis (PRGF®-ENDORET®), plasma with vascular endothelial growth factor (VEGF) was prepared according to the BTI (Spain) protocol. A total of 36 ml of blood divided into four 9 ml test tubes containing 3.8% sodium citrate solution were taken from every patient. After 8 minutes, the samples were centrifuged at 580 g (BTI System IV), and the received plasma was separated into F1 and F2 fractions. Then, 2 ml of the F2 fraction from every test tube were administered to the patients using ultrasound navigation for a perivascular injection and assessment of lesion localization (posterior and anterior tibial arteries).

**Figure 1:**
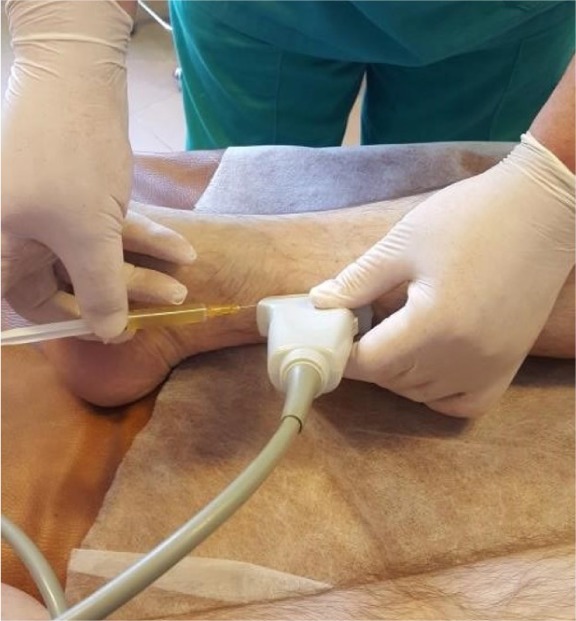
Perivascular VEGF plasma injection (PRGF®-ENDORET®)

Everything was performed in an aseptic environment (Figure 1).

**Figure 2:**
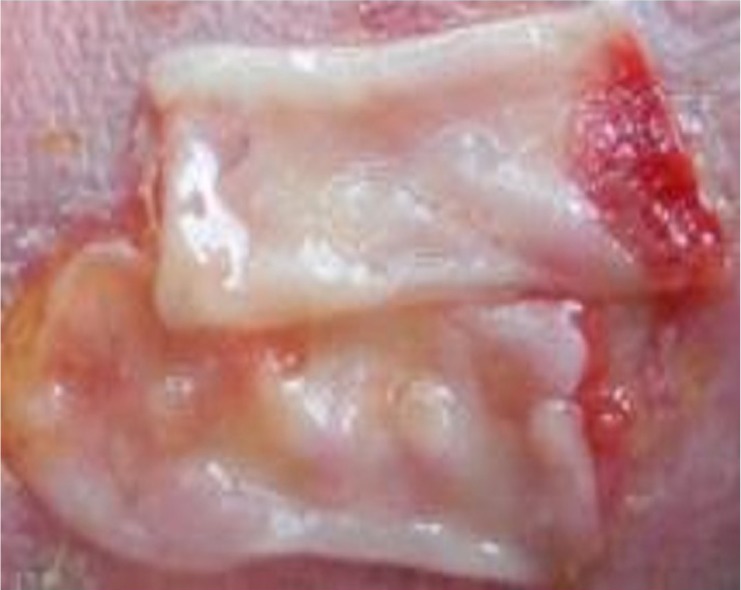
Wound closure using a plasmatic membrane.

After 3-4 days postamputation, the wound area was covered by a plasmatic membrane enriched with growth factors created from F2 (Figure 2).

We used a complex assessment of the blood perfusion after transluminal surgery to opt for wound healing perspectives and spare foot amputation.

According to the angiosomic principle of lower limb vascularization, we measured the temperature on the affected side (electronic thermometer HEACO DT-8806S) on the anteromedial, posteromedial, posterolateral popliteal areas, middle and lower calf levels, medial and posterior areas of the foot before and after transluminal surgery [16]. Transcutaneous oximetry (tcpO2) is also used to assess oxygenation of the lower limb soft-tissue and the microcirculation status. For this, a transcutaneous oximeter from RADIMETER (Denmark) was used. A Clark sensor is placed on the rear of the foot and the calcaneus area. We used the following criteria to evaluate the amount of microcirculation preserved [7]: grade I microcirculation disorder (compensated tissue metabolism) - tcpO2> 30 mm Hg; grade II microcirculation disorder (subcompensated tissue metabolism) - tcpO2 = 20-30 mmHg; grade III microcirculation disorder (decompensated tissue metabolism) tcpO2< 20 mmHg.

Transonic BLF21 (Transonic Systems Inc., NY, USA) was used to measure patients’ tissue perfusion. For these purposes, an R type (right angle) sensor with a 15 mm diameter was used. The sensors are placed using an adhesive ring to two primary areas, soles and back of the foot. BPI, Doppler sonography, objective examination, and patient complaints were all used to evaluate revascularization effectiveness. Rutherford's scale (1997) was used to observe changes in the patient clinical status [17]: 0 – no changes in the ischemia level, no BPI increase); +1 – minimal improvement

(an increase in BPI, more than a 0.1 but without subjective improvement or vice versa); +2 – mild improvement (one point decrease in ischemia, a minimal increase of 0.1 in BPI), +3 – major improvement; Therefore, - 1, - 2, and - 3 stand for minor, mild and major deterioration, respectively.

For the evaluation of the wound regeneration stimulating methods, 3 groups were formed: the first (comparison group) was comprised of 21 patient, who received traditional post-amputation wound care, the second group comprised 29 patients who received revascularization, PRP therapy and regeneration stimulus on days 5-6 of post-amputation treatment in the form of a plasmatic membrane (PRGF®-Endoret®), covering the whole wound area. The control group was comprised of 13 patients with lower limb atherosclerosis before amputation. The first and second groups are representative by age, gender, occlusion level, comorbidities and wound area.

Cytology was gathered on the day of admission and during the 5-6, 8-9, and 12-14 days. The wound healing process was assessed using the imprint cytology. Also, the regenerative-degenerative index was used to evaluate the regeneration and degenerative processes of the lesions.

Cell reactivity was evaluated via TKI (tyrosine kinase activity in relation to tyrosine phosphatase activity) on the 6-10 and 15-day postamputation in order to assess the pathomorphological changes of foot wound healing.

ICC (inter cytokine coefficient) can illustrate the reactivity of the systemic mechanisms that limit altereation and induce proliferation. ICC is a percentage relation of alteration phase cytokines (IL-1β, TNF-α) that increase with the increase in proliferation and remodeling phase cytokines (IF-γ та ІL-4 To determine the interleukin amounts in the plasma, the «Amersham Pharmacia Biotech UK Limited» RIA (radioimmunoassay) reagent set was used. Then, the results were compared to the amount before surgery using the formula listed below.


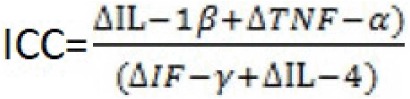


## Results

After analyzing the indicators that allow objectively to assess the blood perfusion quality after revascularization of the distal limb levels, we concluded the indicators for foot amputation and wound healing prognosis.

Thus, effective revascularization is represented by measuring skin temperature on determined the angiosomic areas of the lower limb on the 7 -14 days of the postoperative period (Table 1).

**Table 1:** Skin temperature on angiosomic areas after revascularization.

Measuring areas	Preoperative	7^th^ day after surgery	14^th^ day after surgery
**Mid-tibial level, anteromedial surface**	33.4 ± 0.65	36.1 ± 0.26*	35.9 ± 0.33*
**Lower tibial level, anteromedial surface**	33.7 ± 0.49	35.9 ± 0.38*	38 35.3 ± 0.57*
**Mid-tibial level, posteromedial surface**	33.8 ± 0.56	36.0 ± 0.39*	35.8 ± 0.46*
**Lower tibial level, posteromedial surface**	33.6 ± 0.86	35.9 ± 0.32*	35.4 ± 0.81*
**Mid-tibial level, posterolateral surface**	33.7 ± 0.32	35.9 ± 0.44*	35.6 ± 0.41*
**Lower tibial level, posterolateral surface**	33.5 ± 0.38	35.1 ± 0.34*	35.1 ± 0.42*
**Medial foot surface**	33.3 ± 0.52	35.1 ± 0.17*	34.7 ± 0.44*
**Rear foot surface**	33.2 ± 0.76	34.9 ± 0.12*	34.5 ± 0.56*

Note: * р<0.05comparing to preoperative results.

The absolute tissue perfusion amount on foot soles and rear increased from 0.585 ± 0.109 (ml/min/100g) to 6.77 ± 1.3 (ml/min/100g) and from 0.208 ± 0.086 (ml/min/100g) to 6.77±1.3 (ml/min/100g) (р<0,05) respectively.

A mild and significant improvement (on the Rutherford scale) occurred in 33 (66%) and 17 (34%) of patients regarding the condition of the lower limb (Table 2).

**Table 2:** Transcutaneous oximetry for surgery after the 7^th^ and 14^th^ day after transluminal surgery and PRP-therapy.

tcpO2	N=50		
Pre surgery	7^th^ day after surgery	14^th^ day after surgery
I degree - (tcpO_2_ > 30 mmHg)	-	6	18
II degree - (tcpO_2_ 20-30 mmHg)	12	29	32
III degree - (tcpO_2_ < 20 mmHg)	38	15	-

**Table 3:** Starting cytokine levels (pg/ml) in patients’ plasma.

Group	IL - 1β	IL- 4	TNF – α	IF– γ
Control	807.72 ± 20.59	8.54 ± 0.73	23.46 ± 2.29	45.27 ± 4.15
1^st^	526.60 ± 18.95**	3.77 ± 0.19***	96.87 ± 5.06***	10.41 ± 0.89***
2^nd^	554.32 ± 39.81*	3.56 ± 0.42***	118.80 ± 8.93***	9.76 ± 1.01***

Note: * – p<0.05. ** – p<0.01. *** – p<0.001 comparing to the control group.

According to objective and subjective data, which confirm a significant improvement of limb perfusion, these became the indications for foot amputation. We performed Garanjo's amputations in 5 patients, Shoppar's in 31 and Lisfranc's (or Lisfranc-Hue's) in 14 patients. Postoperative wounds were treated openly due to an insufficient amount of skin flap remaining.

We also analyzed the effect of the plasmatic membrane (PRGF®-Eldoret® supernatant) on the wound regeneration and its preparation for an auto-dermal skin transplant.

Our research has shown that TCI represents cell sensitivity to proliferative processes, migration, and intercellular interactions in damaged tissues and can be informative in wound healing prognosis. However, a more conclusive answer can be made by measuring the cytokine levels because they are responsible for the intercellular interactions and are specific for every phase of the wound healing process [30].

We found that cytokine levels before foot amputation were almost identical in both groups 1 and 2 comparing to the control group. Levels of ІL - 1β, ІL- 4 before amputation were low, while TNF- α levels were 298, 36% (р <0,001), reliably higher in comparison to the control group. The amounts of ІL-1β, ІL-4 and IF-γ were 31.4%, 53.72%, and 71.12% lower compared to the control group (Table 1).

Sometimes, the TNF-α levels were largely elevated in groups 1 and 2 (comparing to the control group by 5.1 and 1.2 times, respectively). The other cytokine amounts were similar. In patients with foot necrosis, ICC was 57.8% lower compared to the control group. This can indicate a metabolic shift in cytokine production tied to the necrotic process.

We compared patients’ ICC, who underwent traditional wound treatment and PRFtherapy. An interesting fact is that ICC in group 1 was raised 3.9 times on average in comparison to pre-amputation levels, mostly due to ІL-1β and TNF-α increase. This can indicate an increase in the alternative and destructive processes in the wound, while low levels of ІL-4 and IF-γ indicate a depletion of proinflammatory reserves. This can be explained by low foot perfusion and ischemia.

ICC dropped lower than initial in patients who underwent foot amputation before revascularization and PRP-therapy, and the wound was closed using a plasmatic membrane. This can be explained by an increase of anti-inflammatory cytokines with a steady number of inflammatory factors. Changes in ІL-4 and IF-γ levels can be considered a transition of the wound healing process to a proliferation phase, while a decrease in ІL - 1β and TNF – α illustrates a diminishing inflammation.

Tyrosine kinase system activity and cytokine interactions represent the alteration and proliferation processes and can be used as objective criteria for an auto-dermal transplant.

Imprint cytology showed a decrease in the number of proinflammatory cells (neutrophils, monocytes, lymphocytes) and an increase in the reparative cells (macrophages and lymphocytes) levels. Therefore, on the second and third days, there was a decrease in the number of neutrophils and lymphocytes and all inflammatory cells on the fourth and fifth days. This shows a transition from a degenerative inflammation to a regeneration. This shift occurred around the eighth day in the majority of patients. The regenerative shift occurred much later, around days 18-21, in patients who received traditional postoperative treatment.

## Discussion

The experience of most vascular surgeons treating necrotic lessons in the distal parts of the foot in patients with CLI shows low effectiveness of the conservative treatment. However, revascularization treatment involves a few technical issues. They are waste and multilevel atherosclerosis of femoral, popliteal and distal segments, which comprise 65-85% of lower limb disease cases, rapid ischemia progression, leading to necrosis and gangrene of the distal parts of the foot, tibial artery occlusion, which excludes bypass surgery, comorbidities, advanced age, which directly influences the revascularization methods and postoperative period, and finally, diabetic foot with predominately distal arterial involvement. All these situations call for transluminal surgery.

One more feature of CLI is a deficiency in the collateral blood flow, leading to a disbalance in blood supply and drainage by the available collateral arteries, and this can become the cause of secondary thrombosis or critical ischemia progression [7]. Many researchers agree on using therapeutic angiogenesis in this situation in combination with endovascular treatment or solely. A collateral growth stimulation. using angiogenic factors such as VEGF, fibroblast growth factor (FGF), transforming growth factor-beta (TGF-β). GM-CSF (granulocyte-macrophage colony-stimulating factor), genetic therapy and stem cells are being discussed [2]. Today, active research on angiogenic factors producing plasmids for managing CLI continues [20-24].

It needs to be said that monotherapy using angiogenic factors did not impress vascular surgeons due to low effectiveness (no reliable lesion closure and physical activity increase. decreases in amputation necessity). The causes of this situation are represented by a rapid degradation of the molecules in the human body. systemic hypotension due to vasodilatation and hemangioma growth after repeated administrations [25-28].

A different genetic construct. with the FGF gene. appeared to be ineffective and had important side effects according to the “TRAFFIC” and “TALISMAN” studies [29-31].

Nevertheless, our research had shown that PRP and PRF are a viable biotechnical direction in managing CLI. We believe that this methods in combination with endovascular surgery is the most effective limb function preserving treatment at the moment. Other benefits are low-cost efficiency, accessibility, no AIDS or hepatitis transfer risk, or immune reactions. Further studying of plasma enriched with thrombocytes effects on the regeneration process is needed to create a practical and theoretical foundation for a new treatment method.

## Conclusion

Endovascular surgery and PRP combination increase distal limb tissue perfusion in CLI patients, which is a good prognostic factor for further postamputation foot healing. Also, the plasmatic membrane enriched with thrombocytes creates optimal conditions for tissue regeneration, thus reducing the time needed for auto-dermal wound closure.

## Conflict of Interest

The authors confirm that there are no conflicts of interest.
